# *Ficus* (Moraceae) and fig wasps (Hymenoptera: Chalcidoidea) in Taiwan

**DOI:** 10.1186/s40529-015-0090-x

**Published:** 2015-05-16

**Authors:** Anthony Bain, Hsy-Yu Tzeng, Wen-Jer Wu, Lien-Siang Chou

**Affiliations:** 1 Institute of Ecology and Evolutionary Biology, College of Life Science, National Taiwan University, No. 1, Sec. 4, Roosevelt Rd.,, Taipei, 10617 Taiwan; 2 Centre d’Ecologie Fonctionnelle et Evolutive CEFE, UMR 5175 CNRS, 1919 route de Mende, Montpellier, 34293 France; 3 Department of Forestry, National Chung-Hsing University, 250 Kuokwang Road, Taichung, 40227 Taiwan; 4 Department of Entomology, National Taiwan University, No. 1, Sec. 4, Roosevelt Rd.,, Taipei, 10617 Taiwan

**Keywords:** Chalcidoidea, Ficus, Fig wasp, Nomenclature, Taiwan

## Abstract

Although *Ficus*-associated wasp fauna have been extensively researched in Australasia, information on these fauna in Taiwan is not well accessible to scientists worldwide. In this study, we compiled records on the *Ficus* flora of Taiwan and its associated wasp fauna. Initial agronomic research reports on *Ficus* were published in Japanese in 1917, followed by reports on applied biochemistry, taxonomy, and phenology in Chinese. On the basis of the phenological knowledge of 15 species of the *Ficus* flora of Taiwan, recent research has examined the pollinating and nonpollinating agaonid and chalcid wasps (Hymenoptera: Chalcidoidea). Updating records according to the current nomenclature revealed that there are 30 taxa (27 species) of native or naturalized *Ficus* with an unusually high proportion of dioecious species (78%). Four species were observed to exhibit mutualism with more than one pollinating wasp species, and 18 of the 27 *Ficus* species were reported with nonpollinating wasp species. The number of nonpollinating wasp species associated with specific *Ficus* species ranges from zero (*F. pumila*) to 24 (*F. microcarpa*). Approximately half of the Taiwanese fig tree species have been studied with basic information on phenology and biology described in peer-reviewed journals or theses. This review provides a solid basis for future in-depth comparative studies. This summary of knowledge will encourage and facilitate continuing research on the pollination dynamics of *Ficus* and the associated insect fauna in Taiwan.

**Electronic supplementary material:**

The online version of this article (doi:10.1186/s40529-015-0090-x) contains supplementary material, which is available to authorized users.

## Review

### Introduction

The pantropical genus *Ficus* (Moraceae) is the most speciose genus of woody plants, comprising 735 species known worldwide (Berg and Corner [2005]). *Ficus* is characterized by their unique inflorescences, called figs, or syconia. Due to their essential role in tropical landscapes and their rich ecological relationships with numerous invertebrates and vertebrates, fig trees may be considered as keystone resources of tropical forests (Shanahan et al. [2001]; Harrison [2005]). Asia contains a wide diversity of *Ficus* flora, with 130 known species from Borneo (Berg and Corner [2005]), 99 from China (Wu et al. [2003]) and only 25 species common to the two areas.

The genus has attracted considerable attention among ecologists because of its obligate mutualism with pollinating wasps (Hymenoptera: Agaonidae: Agaoninae, Kradibiinae, Tetrapusiinae) (Cruaud et al. [2010]; Heraty et al. [2013]). Fig trees have become an essential model for studies on mutualism (Janzen [1979]; Frank [1985]), sex ratio theory (Herre [1985]; Weiblen [2002]), and coevolution processes (Anstett et al. [1997]; Cook and Rasplus [2003]).

Fig trees and their pollinators have long been used as an example of obligate mutualism. The pollinating wasps are the only organism pollinating the figs and these wasps can only lay eggs in fig ovules. The pollinators enter into the fig by a tight ostiole. Once inside the fig, wasps pollinate the flowers and lay eggs inside the fig ovules (Kjellberg et al. [2005]). Then the larvae feed on gall tissue induced during the oviposition and mature along with the seeds and pollen grains of the fig. At maturity, fertilized female pollinating wasps leave the natal fig and transport pollen to another receptive fig on another tree (Kjellberg et al. [2005]). Some pollinating fig wasps genera actively pollinate the styles of the ovules before oviposition. After mating, they open the anthers of their natal fig and collect pollen grains that are stored in pollen pockets located on the ventral side of the mesosoma (Kjellberg et al. [2001]). In contrast, passive pollination requires no specific behavior: The pollen grains simply stick to the wasp body and fertilize ovules when the pollinating wasps enter a fig. Though one *Ficus* species is associated with only one pollinating wasp species in most of the cases (Janzen [1979]), some *Ficus* species have long been known to host additional pollinating wasp species (Galil and Eisikowitch [1967]; Ramírez [1970]; Molbo et al. [2003]).

In addition to the pollinating wasp species, most of the *Ficus* species also host nonpollinating wasp species (Kjellberg et al. [2005]). These wasps oviposit from the outside of the figs. The number of nonpollinating wasps (NPFW) species varies greatly between *Ficus* species (Kerdelhué et al. [2000]). Their feeding regimes also vary: Some NPFW species gall the ovules similarly to the pollinating species, and some are parasitoids (Compton and van Noort [1992]).

**Figure 1:**
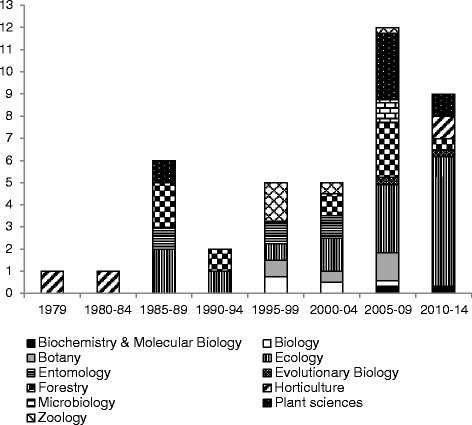
Taiwanese publications on fig and fig wasps since 1979. Each Taiwanese article has been classified under a discipline in which the journal they have been published in. The categories are the ones used in the ISI Web of Knowledge^SM^. Nevertheless 18 of the 24 cited journals were not referenced by ISI Web of Knowledge^SM^ then they have been categorized according to the journal description. For the journals having more than one category, value has been divided in equal parts. For example, a journal categorized in Forestry and Ecology would have counted as 0.5 in the two categories for this graph.

Over the past century, extensive research on various characteristics of the fig flora in Taiwan and its associated fauna has been conducted (Figure 1). Taiwan and its offshore islands are tropical and subtropical. Recently, 26 native and one introduced *Ficus* species have been reported (Tzeng [2004], in Chinese with English abstract). The first report on the *Ficus* genus in Taiwan was written in Japanese and focused on the cultivation of *Ficus pumila* var. *awkeotsang* (Takao [1917]). The first taxonomic monograph was published in 1934 (Sata [1934]), followed 10 years later, by a comparison of the fig flora in Taiwan and in the Philippines (Sata [1944]). Later on, several studies addressing the biochemistry of an edible jelly produced from the dried seeds of *F. pumila* var. *awkeotsang* (Huang and Chen [1979]; Huang et al. [1980]; Lin et al. [1989]; Liu et al. [1989], [1990]) were published. This jelly, locally called “aiyu”, is a common ingredient of summer beverages in Taiwan.

Taxonomic research on the *Ficus* genus in Taiwan resumed 45 years after Sata’s final publication (Liao [1989]), which has been recently updated by Tzeng ([2004]). The research performed by Tzeng ([2004]) is exhaustive and provides a clear understanding of the *Ficus* flora and its distribution around Taiwan.

The phenology of *Ficus* has been extensively studied (Hu et al. [1986]; Ho [1987]). Phenological research introduced a physiological point of view to the study of fig ecology. However, common fig-wasp interactions have rarely been reported in Taiwan. We here establish a framework for future work on Taiwan *Ficus* and its associated wasp fauna by providing an updated list of *Ficus* species and the associated wasp species in Taiwan. These wasp species include specific pollinators or groups of pollinator species (in a few cases) as well as nonpollinating fig wasps.

As part of a previous field study (Bain [2012]), we collected figs from various sites throughout a lowland forest habitat on Taiwan as well as on Orchid and Green Islands off the southeast coast of Taiwan.

### Notes on the taxonomy of *Ficus*(Moraceae)

In this present review, historical records were updated according to the current taxonomy and nomenclature guidelines to list 27 fig species (one species more than previously recorded) and 30 distinct taxa associated with the six subgenera present in Taiwan: *Urostigma* (5 taxa), *Pharmacosycea* (2), *Ficus* (8), *Synoecia* (6), *Sycidium* (6), and *Sycomorus* (3) (Table 1).

**Table 1:** **Taiwan** ***Ficus*** **taxa and their associated pollinating and non-pollinating wasp species**

***Ficus***	Fig wasps	References
Subgenus *Urostigma* (monoecious)		
*F. benjamina* L. var. *bracteata* Corner	*Eupristina koninsbergeri*^P^*	Grandi [1916]
	*Philotrypesis distallatoria*	Grandi [1926]; Chou and Wong [1997]
	*Micranisa* sp1 Sycoryctini sp.	Bain A. unpublished data; Segar et al. [2012]
*F. caulocarpa* Miq.	*Platyscapa fischeri*^P^*	Wiebes [1977]; Yokoyama and Iwatsuki [1998]
	*Platyscapa hsui* ^P^	Chen and Chou [1997]
	*Camarothorax* sp2*	Yokoyama and Iwatsuki [1998]
	Otitesella clarae*	Wiebes [1977]; Yokoyama and Iwatsuki [1998]
*F. microcarpa* L. f.	*Eupristina verticillata* ^P^	Waterston [1921]; Chen and Chou [1997]
	*Walkerella kurandensis*	Bouček [1988]; Chen et al. [1999]
	*Walkerella microcarpae*	Bouček [1993]; Yang H-W unpublished data
	*Acophila microcarpa*	Chen et al. [1999]
	*Bruchophagus sensoriae*	
	*Meselatus bicolor*	
	*Micranisa degastris*	
	*Ormyrus lini*	
	*Philotrypesis taiwanensis*	
	*Sycophila curta*	
	*Sycophila maculafacies*	
	*Sycophila petiolata*	
	*Sycoryctes moneres*	
	*Odontofroggatia quinifuniculus*	Feng and Huang [2010]; Yang H-W unpublished data
	*Philotrypesis emeryi*	Grandi [1926]; Chen et al. [1999]
	*Eufroggattisca okivanensis*	Ishii [1934]; Chen et al. [1999]
	*Odontofroggatia gajimaru*	
	*Philotrypesis okinavensis*	
	*Sycoscapter gajimaru*	
	Micranisa yashiroi*	Ishii [1934]; Yokoyama and Iwatsuki [1998]; Beardsley [1998]
	*Conidarnes* sp1	Cruaud et al. [2011]
	*Odontofroggatia corneri*	Wiebes [1980]; Chen et al. [1999]
	*Odontofroggatia galili Odontofroggatia ishii*	
*F. subpisocarpa* Gagnep.	*Platyscapa ishiiana* ^P^	Grandi, [1923]; Chen and Chou, [1997]
subsp. *subpisocarpa* Corner	*Otitesella ako*	Ishii, [1934]; Bain A. unpublished data
	*Acophila mikii*	
	*Philotrypesis* sp1*	Yokoyama and Iwatsuki [1998]
	*Sycoscapter* sp1*	
	*Camarothorax* sp1*	
	*Camarothorax* sp2, sp3	Bain A. unpublished data
*F. subpisocarpa* Gagnep.	*Micranisa* sp1	Bain A. unpublished data
subsp. *subpisocarpa*	*Ormyrus* sp1, sp2	
Corner	*Philotrypesis* sp1	
	*Sycophila* sp1, sp2, sp3, sp4, sp5	
	*Walkerella* sp1	
	*Arachonia* sp1	Cruaud et al. [2011]; Segar et al. [2012]; Bain A. unpublished data
	*Sycoryctes* sp1	
	*Sycoscapter* sp2	
*F. religiosa* L.	*Platyscapa quadraticeps* ^P^	Mayr [1885]; Chen and Chou [1997]
	Otitesella digitata*	Westwood [1883]; Wiebes [1966]
	Otitesella religiosa*	
	Sycoscapter gracilipes*	
	Sycoscapteridea monilifera*	
	Philotrypesis anguliceps*	Westwood [1883]; Wiebes [1966]; Bouček [1988]
Subgenus *Ficus* (dioecious)		
*F. erecta* Thunb. var. *beecheyana* King	*Blastophaga nipponica* ^P^	Grandi [1921]; Chen and Chou [1997]
	*Sycoscapter inubiae,* sp1	Ishii [1934]; Tzeng et al. [2006b], Tzeng et al. [2008]
*F. formosana* Maxim.	*Blastophaga taiwanensis* ^P^	Chen and Chou [1997]
	*Sycoscapter* sp1, sp2	Tzeng et al. [2008]
*F. pedunculosa* Miq. var. *pedunculosa*	*Blastophaga peducunlosae* ^P^	Chen and Chou [1997]
	(Parasitic fauna unknown)	
*F. pedunculosa* Miq. var. *mearnsii* Corner	*Blastophaga peducunlosae* ^P^	Chen and Chou [1997]
	*Apocrypta* sp.	Bain, unpublished data
*F. ruficaulis* Merr.	*Valisia filippina* ^P^	Wiebes [1993]; Chen and Chou [1997]; Cruaud et al. [2010]
	(Parasitic fauna unknown)	
*F. tannoensis* Hay.	*Blastophaga tannoensis* ^P^	Chen and Chou [1997]
*F. triloba* Buch.-Ham. subsp. *triloba* Corner	*Valisia esquirolianae* ^P^	Chen and Chou [1997]; Cruaud et al. [2010]
	Sycoryctini sp.	Segar et al. [2012]; Bain A. pers. obs.
*F. vaccinoides* Hemsl.	*Blastophaga yeni* ^P^	Chen and Chou [1997]
	(Parasitic fauna unknown)	
Subgenus *Pharmacosycea* (monoecious)		
*F. nervosa* subsp. *nervosa* Heyne	*Dolichoris nervosae nervosae* ^P^ *	Hill [1967]
	*Philotrypesis* sp1	
	*Sycoscapter* sp1	
*F. nervosa* subsp *pubinervis* Blume	*Dolichoris valentine*^P^*	Grandi [1916]
	(Parasitic fauna unknown)	
Subgenus S*ynoecia* (dioecious)		
*F. pumila* L. var. *pumila*	*Wiebesia pumilae* ^P^	Hill [1967]; Chen and Chou [1997]
	*Wiebesia* sp.^P^	Wang et al. [2013]
	No parasitic wasp in Taiwan	
*F. pumila* L. var. *awkeotsang* Corner	*Wiebesia pumilae* ^P^	Hill [1967]
	*Wiebesia* sp.^P^	Wang et al. [2013]
	No parasitic wasp in Taiwan	
*F. punctata* Thunb. f. *aurantiacea* Corner	*Wiebesia contubernalis* ^P^	Grandi [1927a]; Chen and Chou [1997]
	*Sycoscapter* sp.	Chou and Yeh [1995]
*F. sarmentosa* Buch.-Ham.	*Wiebesia callida* ^P^	Grandi [1927a]; Chen and Chou [1997]
var. *nipponica* Corner	(Parasitic fauna unknown)	
*F. trichocarpa* Blume	*Wiebesia vechti*^P^*	Wiebes [1993]
	(Parasitic fauna unknown)	
Subgenus S*ycidium* (dioecious)		
*F. ampelas* Burm. f.	*Krabidia sumatrana* ^P^	Wiebes [1993]; Chen and Chou, [1997]
	*Philotrypesis distallatoria*	Grandi [1926]; Chang [2003]
	*Philotrypesis jacobsoni*	Grandi [1926]; Chou and Wong [1997]
	*Sycoryctes* sp1, sp2	Chang [2003]
	Epichrysomallinae sp.	
*F. cumingii* Miq.	*Krabidia panchoi* ^P^	Wiebes [1993]; Chen and Chou [1997]
	(Parasitic fauna unknown)	
*F. heteropleura* Blume	*Krabidia dubium*^P^*	Grandi [1926]; Cruaud et al. [2010]
	(Parasitic fauna unknown)	
*F. irisana* Elm.	*Krabidia commuta* ^P^	Wiebes [1993]; Chen and Chou [1997]
	*Herodotia* sp.	Chen [1998]
	*Philotrypesis* sp1, sp2	
	*Sycophila* sp1, sp2	
	*Sycoscapter* sp.	
*F. tinctoria* G. Forst. subsp. *swinhoei* King	*Krabidia gibbosae* ^P^	Hill [1967]; Chen and Chou [1997]; Cruaud et al. [2010]
	*Neosycophila* sp.	Huang [2007]
	*Philotrypesis* sp1, sp2	
	*Sycoscapter* sp.	
	*Eufroggatisca* sp.	Tzeng H-Y unpublished data
	*Sycoryctes* sp.	
	*Sycophila* sp.	
*F. virgata* Reinw.	*Krabidia philippinensis* ^P^	Hill [1969]; Chen and Chou [1997]; Cruaud et al. [2010]
	*Krabidia virgatae*^P^*	Hill [1969]; Cruaud et al. [2010]
	*Krabidia sessilis*^P^*	
*F. virgata* Reinw.	*Camarothorax* sp.	Bain A. unpublished data
	*Philotrypesis* sp.	
	Sycoryctini sp.	Segar et al. [2012]; Bain A. unpublished data
Subgenus S*ycomorus* (dioecious)		
*F. benguetensis* Merr.	*Ceratosolen wui* ^P^	Chen and Chou [1997]
	*Philotrypesis* sp1, sp2	Bain A. unpublished data
	*Sycoscapter* sp1, sp2	
*F. septica* Burm.	*Ceratosolen bisulcatus bisulcatus*^P^*	Mayr [1885]; Wiebes [1994]
	*Ceratosolen bisulcatus jucundus* ^P^	Grandi [1927b]; Wiebes [1994]; Lin et al. [2011]
	*Ceratosolen* sp.^P^	
	*Sycophaga* sp.*	Bain A. unpublished data; Cruaud et al. [2011]
	*Philotrypesis* sp1, sp2	Ho [2009]
	*Sycoscapter* sp.	
	*Philotrypesis spinipes*	Mayr [1885]; Chou and Wong [1997]
	Philotrypesis bimaculata*	
*F. variegata* Blume	*Ceratosolen appendiculatus* ^P^	Mayr [1885]; Chen and Chou [1997]
	Apocrypta caudata*	Weiblen et al. [1995]
	Sycophaga spinitarsus*	Mayr [1885]; Rasplus J.-Y. pers. obs.
	Sycoscapter patellaris*	(www.figweb.org/Fig_wasps/Faunal_assemblages/Indo-Australasia/China); Cruaud et al. [2011]

The pollinating wasps are noted with a superscripted p whereas the wasp species not yet observed in Taiwan but reported elsewhere for these fig taxa are noted with an asterisk (*). The last column displays the references of the description, the name modifications of the given species and/or the observations on these species.

Several species names from the studies of Liao ([1989], [1995]) and Tzeng ([2004]) were updated according to the recent taxonomic and nomenclatural knowledge. In the subgenus *Urostigma*, *F. subpisocarpa* has been subject to two recent revisions. Berg and Corner ([2005]) reinstated the species from *F. superba* var. *japonica*. Subsequently, by incorporating new observations from Thailand, Berg further divided the species into two subspecies: *F. subpisocarpa* subsp. *pubipoda* and *F. subpisocarpa* subsp. *subpisocarpa* (Berg [2007]). Based on this knowledge, the Taiwanese taxon is *F. subpisocarpa* subsp. *subpisocarpa* (hereafter called *F. subpisocarpa*). Several taxonomic questions for this subgenus group remain unanswered. For example, the taxonomic position of *F. benjamina* var. *bracteata* is unclear. In 1983, Yamazaki described *F. benjamina* var. *bracteata* from Taiwan for the first time; subsequently, Berg and Corner ([2005]) assigned it a synonym: *F. benjamina*. In the studies conducted by Berg and Corner ([2005]) and Corner ([1965]), the analyzed *F. benjamina* var. *bracteata* samples were not obtained from Taiwan. Our observations from southern Taiwan reveal differences between *F. benjamina* var. *benjamina* and *F. benjamina* var. *bracteata* (Bain and Tzeng, pers. obs.). Despite these differences, until further research provides a new basis for a decision, we continue to list *F. benjamina* var. *bracteata* as a variety according to descriptions provided by Tzeng ([2004]). In addition, *F. religiosa* was not listed as a native species in the report of Sata ([1934]), but as introduced to Taiwan. Nevertheless, because the pollinating wasp species of *F. religiosa*, *Platyscapa quadraticeps*, has been observed in Taiwan, we consider *F. religiosa* a naturalized species (Chen and Chou [1997]).

Two previously reported species of the subgenus *Pharmacosycea* from Taiwan have been reported (*F. nervosa* subsp. *nervosa* and *F. nervosa* subsp. *pubinervis*) have a debatable taxonomic status. Tzeng ([2004]) considered the aforementioned species as two distinct species whereas Berg and Corner ([2005]) listed them as subspecies. Both species are allopatric: *F. nervosa* subsp. *nervosa* is distributed in southern Taiwan and *F. nervosa* subsp. *pubinervis* is distributed only in Orchid Island (the island, located offshore on the Southeast of Taiwan Island, is also called Lanyu, 22°03’N; 121°32’E) (Tzeng [2004]). The fact that they are pollinated by different agaonid wasp species (Table 1) provides additional evidence for distinguishing them as different species. According to pollen, pyrena and leaf morphology evidence (Chuang [2000]; Tzeng [2004]; Tzeng et al. [2009]), *F. nervosa* subsp. *nervosa* and *F. nervosa* subsp. *pubinervis* are phylogenetically close, yet distinct species. Thus, in this study, we refer to *F. nervosa* subsp. *nervosa* and *F. nervosa* subsp. *pubinervis* as *F. nervosa* and *F. pubinervis*, respectively.

In the subgenus *Sycidium*, *F. tinctoria* subsp. *swinhoei* has been synonymized under *F. tinctoria* subsp. *tinctoria* (Berg and Corner [2005]). The distribution of the former taxon is limited to southern Taiwan and Orchid Island, whereas *F. tinctoria* is widely distributed throughout Australasia (Berg and Corner [2005]). Moreover, these two subspecies have different pollinators (J-Y Rasplus, pers. obs.). Therefore, on the basis of the study by Tzeng ([2004]), we continue to list *F. tinctoria* subsp. *swinhoei* as separated from *F. tinctoria* subsp. *tinctoria. Ficus tinctoria* and *F. virgata* are two species that require further taxonomic investigation. After solely studying herbarium samples, Berg and Corner ([2005]) could not clearly distinguish between Taiwanese *F. tinctoria* and *F. virgata*. However, according to local field observations, *F. virgata* can be clearly and unambiguously distinguished from other *Ficus* species (Liao [1989], [1995]; Tzeng [2004]). Thus, Taiwanese *F. virgata* and *F. tinctoria* subsp. *swinhoei* are here considered distinct species.

Furthermore, in the subgenus *Ficus*, *F. esquiroliana* has been synonymized under *F. triloba* subsp*. triloba* (Berg [2007]). We support this decision because we found morphologically similar trees in Yunnan, China, and Taiwan (Bain and Tzeng, pers. obs.).

In addition, *F. benguetensis* (subgenus *Sycomorus*) has been reinstated as a full species (Tzeng [2004]; Berg and Corner [2005]). Previously, *F. benguetensis* was considered a variety, *F. fistulosa* var. *benguetensis* (Liao [1989], [1995]); in addition, Berg ([2011]) amended its description.

Finally, *F. aurantiacea* var. *parvifolia* (subgenus *Synoecia*) has been synonymized under *F. punctata* (Berg and Corner [2005]). Two forms of *F. aurantiacea* var. *parvifolia* have been described. The taxon distributed in Taiwan is listed under the “*aurantiacea* form” (i.e., *F. punctata* f. *aurantiacea*) (Chou and Yeh [1995]).

Morphological studies have facilitated the confirmation of the classification of Taiwanese *Ficus* (Shieh [1964]; Chuang [2000]; Tseng et al. [2000]; Bai [2002]; Chuang et al. [2005]; Chang et al. [2009]; Tzeng et al. [2001], Tzeng et al. [2005b], Tzeng et al. [2006a], Tzeng et al. [2009]). Among these studies, pollen (Tzeng et al. [2009]) and pyrena (Chuang [2000]; Chuang et al. [2005]; Tzeng et al. [2006a]) morphologies were interpreted systematically. For example, the morphology of pyrena (fig seed) is different for each *Ficus* subgenus. Moreover, the rough surface of the *Ficus* from the subgenus *Sycomorus* can be linked with their dispersers: Fruit bats (Lee et al. [2009]). Pollen shape lends insight into pollination patterns. Emarginate-ellipse and truncate-ellipse pollen types indicate passive pollination, whereas the truncate-rhombus pollen type indicates active pollination (Kjellberg et al. [2001]).

### Phenology, ecology, and biology of figs and fig wasps

*Ficus* ecology, particularly the interspecific mutualism between *Ficus* and fig wasps, began to receive attention in the early 1990s. Since then, several studies on this interspecific mutualism have been conducted (see Kjellberg et al. [2005] for review).

Prior knowledge of phenology is essential for studies on mutualism. *Ficus* trees differ from most of other tree species: the figs they produced host their mutualistic pollinators. Thus the *Ficus* reproductive phenology is not constant as other tree species (Bain et al. [2014a]) that are, for example, bound to seasons (spring bloom). Numerous phenological studies of *Ficus* trees have been conducted in Taiwan. The subgenus *Urostigma* includes monoecious taxa, whereas all other subgenera in Taiwan are dioecious, having separate male and female trees. The latter produce only seeds whereas the figs of the former produce both pollen and pollen dispersers (pollinating fig wasp). Among the six subgenera in Taiwan, phenological data on all subgenera, except for the subgenus *Pharmacosycea*, have been collected. Finally, among the 30 *Ficus* taxa, only half of them have seen their phenology examined. The most studied taxon is *F. erecta* var. *beecheyana*, which has been described in six reports. In Taiwan, most phenological research has been undertaken as a part of graduate thesis work, and, therefore, is found mainly in Chinese language theses and remains unpublished in peer-reviewed journals. Nevertheless, data from this phenological research provides a strong basis for further study.

The monoecious *F. microcarpa* has been a study subject of four theses in Taiwan (Hsieh [1992]; Chen [1994]; Chen [2001]; Yang [2011]). *Ficus microcarpa* is the most studied species worldwide because of its common occurrence in cities and campuses, and its invasive status in several continents (Beardsley [1998]; Farache et al [2009]; Doğanlar [2012]). Reports on fig production are in agreement with the aforementioned studies. Each of these three studies surveyed the *F. microcarpa* population on the National Taiwan University campus in different years. Fig trees were found to bear figs almost constantly throughout the year, with a decrease in fig yield observed from the beginning of autumn (Hsieh [1992]; Yang et al. [2013]) and some years, no figs were observed on the trees (Chen et al. [2004]). In all of the aforementioned studies, the fig yield was the lowest in the winter season. Moreover, the number of crops per year varied greatly from zero to four. In addition, fig bearing in the *F. microcarpa* population was highly asynchronous as no distinct seasonal or annual pattern was identified in any of the studies. However, the genetic diversity seems to determinate the phenological diversity of the *F. microcarpa* trees (Yang et al. [2014]).

In addition to *F. erecta*, numerous dioecious species have been surveyed to determine fig production patterns (Tzeng et al. [2003], [2005a]; [2006b]; Bain et al. [2014a]). In northern Taiwan, dioecious species were found to have similar phenological patterns across the genus. First, male trees consistently began bearing figs at the beginning of spring every year; female trees began their fig productiona few weeks later. Second, a noticeable second production peak occurred in September and October. Third, rarer winter figs have a longer maturation period. Similar to the *F. microcarpa* population, other fig tree populations produced figs asynchronously. Although there was a peak production period, the production of figs was not simultaneous. The production of some trees can be delayed for a few weeks (Yao [1998]; Bain et al. [2014a]). After the spring crop, the populations bore a low number of figs until autumn, when the male trees again preceded the female trees with a production few weeks earlier. Finally, in winter, the trees were barest throughout Taiwan (Ho [1991]; Chen [1998]; Yao [1998]; Chang [2003]; Huang [2007]; Ho et al. [2011]; Chen [2012]; Chiu [2012]; Bain et al. [2014a]).

Nevertheless, some inter- and intraspecific variations were observed. The duration of the spring crop and the proportion of male trees producing figs between the two crop peaks differed between species. Also the synchrony between male and female tree peaks of fig production varied greatly. Throughout most of the island of Taiwan, *Ficus* tree populations were found to crop during a long period in spring. A considerable proportion of male trees produce figs throughout the year (Ho [1991]; Chen [1998]; Yao [1998]; Chang [2003]; Huang [2007]; Ho et al. [2011]; Chen [2012]; Chiu [2012]; Bain et al. [2014a]). At low altitudes in the extreme north, male trees have shorter spring crops than those in the south. The percentage of male trees producing figs between the two peak seasons is lower in the north than that in the south of Taiwan (Bain et al. [2014a]). Finally, at higher altitudes, the production peaks of male trees during spring are extremely short and are synchronous within a population, with few male trees bearing figs between the two seasonal peaks (Wu [1996]; Tzeng et al. [2003], [2006b]; Bain et al. [2014a]). The general island-wide pattern suggests that phenology has been shaped by environmental factors but constrained by mutualism: the short lifespan of the pollinating fig wasps requires the fig tree population to produce figs regularly (Bain et al. [2014a]). Under harsh environmental conditions, male trees can produce figs only during a short period as soon as spring conditions permit, whereas under mild environmental conditions, the fig production period is extended.

In Taiwan, biochemical studies on *Ficus* have centered on *F. pumila* var. *awkeotsang*, which produces an edible jelly, and the two varieties (var. *pumila* and var. *awkeotsang*) with morphological features have drawn research attention. The two varieties are morphologically close (Lin et al. [1990]; Tzeng [2004]). Because of the economic interest in the edible jelly produced from the dried seeds of *F. pumila* var. *awkeotsang*, the biochemical and nutritional composition of the jelly has been established (Huang and Chen [1979]). Later, the compounds responsible for the jelly have been identified (Liu et al. [1990]). The vegetative reproduction characteristics of this species have also been reported (Liu et al. [1989]).

### Pollinating fig wasps (Hymenoptera: Agaonidae)

According to the phylogenetic nomenclature of Cruaud et al. ([2010]), we have noticed two changes in the former Taiwanese fig wasp nomenclature. First, the wasps belonging to the genus *Blastophaga* subgenus *Valisia* have been listed under the new genus *Valisia*. Therefore, pollinators of *F. triloba* and *F. ruficaulis* are now known as *Valisia esquirolianae* and *V. filippina*. Second, the genus *Liporrhopalum* has been synonymized under the genus *Krabidia*. Thus, all Agaonidae wasps pollinating the *Ficus* species from the subgenus *Sycidium* have been moved to the genus *Krabidia* (Table 1).

The study by Chen and Chou ([1997]) was one of the few studies that attempted to describe all pollinating wasp species from Taiwan. In their study, 24 species (seven newly described species) from eight genera were observed in Taiwan (Chen and Chou [1997]). Their study still observed the 1:1 species specificity rule between fig trees and pollinating wasps. However, recently, a genetic study on the pollinating wasp species of *Ficus septica* concluded that it has three pollinator species with different distributions in Taiwan (Lin et al. [2011]). One species was strictly limited to Orchid Island and the extreme south of Taiwan. The second species was limited only to Orchid Island and was considered rare. The third species was widely observed throughout Taiwan. Furthermore, genetic results showed weak differentiation among the fig wasp populations on the island, suggesting that the gene flow is high within the *F. septica* population in Taiwan (Lin et al. [2008]). This trend was previously observed in other *Ficus* species, fig wasps, and other locations (Compton et al. [2000]; Harrison and Rasplus [2006]; Ahmed et al. [2009]; Kobmoo et al. [2010]). In addition, *Wiebesia pumilae* and *Wiebesia* sp., the pollinators of *F. pumila* var. *pumila* and *F. pumila* var. *awkeotsang*, were morphologically and genetically distinct (Lee [2009]; Jiang [2011]). These two wasp species have been observed in the figs of both varieties of *F. pumila* (Lu et al. [1987]; Jiang [2011]).

In addition to taxonomic studies, since the late 1990s, studies on the population dynamics of pollinators associated with *Ficus* phenology have been conducted (Chen et al. [2004]). The most recent phenological study on *F. microcarpa* in Taipei City provided data on the size of the pollinating wasp population (Yang et al. [2013]). The population size varied greatly during a year. During winter, the pollination rate of figs was low whereas in summer the size of the pollinating wasp population was great and the number of foundresses could reach 19 in one single fig. These data have been used to estimate the total population of female wasps living around the studied group of *F. microcarpa* trees in Taipei (Yang et al. [2013]). Yang et al. ([2013]) showed marked variation in the dynamics of the foundress population size from 0 to 40,000 within one season for the 29 studied trees. Although there was a winter trough in the number of pollinators, the pollinator population could exhibit a high recovery rate in the spring season and still reach the peak during the summer-fall season.

### Nonpollinating fig wasps (Hymenoptera)

Nonpollinating fig wasps (NPFWs) are categorized in three trophic categories: the gallers that induce a gall from the plant tissue, their larva feeds on the growing gall tissue; the parasitoids that lay their eggs on other larvae which feed on the host larva; and the kleptoparasites that kill galler larvae to feed on the induced gall tissues.

The NPFWs belong to three families (Eurytomidae, Ormyridae, and Torymidae) and seven subfamilies (Colotrechninae, Epichrysomallinae, Otitesellinae, Pteromalinae, Sycoecinae, Sycophaginae, and Sycoryctinae). The recent molecular phylogeny of the superfamily Chalcidoidea (Munro et al. [2011]), which includes all of the aforementioned groups, has shown that four groups are monophyletic (Agaonidae, Epichrysomallinae, Pteromalinae, and Sycophaginae), whereas the other groups are paraphyletic. In addition, phylogenies of the subfamilies Sycophaginae (Cruaud et al. [2011]) and Sycoryctinae (Segar et al. [2012]) have been established. As we previously modified the names of pollinating wasp species, we here display the names of the Taiwanese species on the basis of the recent updates (Cruaud et al. [2011]; Segar et al. [2012]). First, the genus *Apocryptophagus* forms a single taxon with the genus *Sycophaga*, and consequently, it has been considered a junior synonym of *Sycophaga* and then synonymized under the genus *Sycophaga* (Cruaud et al. [2011]). Therefore, the former *Apocryptophagus* wasps are currently named *Sycophaga*. Second, the *Sycoscapter* wasps once formed a group that was synonymized by Bouček ([1988]), all of the former names were reinstated by Segar et al. ([2012]): *Sycoscapter*, *Sycoryctes*, *Arachonia*, *Sycoscapteridea*, and *Sycorycteridea*. Nevertheless, some *Sycoscapter* wasps listed in Table 1 and cited from other studies may be still grouped under *Sycoscapter sensu* Bouček ([1988]).

The first and only taxonomic publication on Taiwanese NPFW addressed the *F. microcarpa* wasp community (Chen et al. [1999]). Studies examining NPFWs have been ecological studies, such as a study of the feeding regime (galler or parasitoid) of some *Sycoscapter* larvae (Tzeng et al. [2008]). Conversely, the ecology of Taiwanese NPFW has been thoroughly studied. First, regarding *F. microcarpa*, to determine whether some NPFW are galler species (gallers produce plant galls that contain a growth of tissue to feed their larvae), the fig ostiole (i.e., the only entry of the fig) was sealed to avoid the entry of the pollinating wasps (Chen et al. [2001]). Without the agaonid wasps, two NPFW species laid eggs inside the fig ovules from the outside: *Odontofroggatia* sp. (Epichrysomallinae) and *Walkerella kurandensis* (Otitesellinae). Chen et al. ([2001]) showed that these two species were undoubtedly gallers. Second, regarding *F. formosana*, the exclusion of the two *Sycoscapter* species showed that they had a negative effect on the pollinating wasp population (Tzeng et al. [2008]). In another study, Tzeng et al. ([2014]) showed that the fig wall thickness is a factor affecting the NPFW oviposition. Moreover, the timing of oviposition of these NPFW clearly indicated that the wasps were parasitoids.

Recent observations have shown that the NPFW species occurring on *F. pedunculosa* var. *mearnsii* belong to the genus *Apocrypta* (Bain, unpublished data). This genus was reported to feed on the larvae of pollinating wasps from the genus *Ceratosolen* (Ulenberg [1985]), all pollinators of the fig subgenus *Sycomorus* (Rønsted et al. [2005]). However, *F. pedunculosa* var. *mearnsii* belongs to the subgenus *Ficus* and is pollinated by *Blastophaga* wasps, but not by *Ceratosolen* wasps. Therefore, this observation is unexpected and should be further confirmed by studying more trees and by covering a larger area.

Finally, NPFWs are the prey of numerous ant species (Formicidae). Such ant species have been observed foraging inside figs of *F. tinctoria* subsp. *swinhoei*, *F. septica*, *F. benguetensis*, and *F. subpisocarpa* (Bain et al. [2014b]). Ants enlarged the wasp exit hole and entered inside the figs to prey on the remaining fig wasps. On *F. subpisocarpa*, ants live more closely on the tree nesting inside the living branches of the tree (Bain et al. [2012]). In these nests, numerous bodies of nonpollinating and pollinating wasps have been collected. Nevertheless, the foraging and hunting behaviors of the ants seem to be species dependent as wasp bodies have not been found in the nests of every ant species (Bain et al. [2012]).

## Conclusion

This paper presents and organizes the abundant and previously difficult-to-access research data on *Ficus* species and fig wasps in Taiwan. This paper compiles data from internationally accessible English language journal articles as well as local theses and dissertations, mostly in Chinese. In addition, this paper includes data from recent research conducted by the authors of this paper and presents an elaborate picture of the insect communities living on fig trees. The number and diversity of fig wasp fauna as well as the wide taxonomical range of *Ficus* warrant further comparative studies on the insect communities. Moreover, the high proportion of dioecious species enables investigating the sexual differences and adaptations of the two sexes. In summary, this paper provides comprehensive information on *Ficus* flora and wasp fauna in Taiwan, establishing a basis for understanding fig wasp survival and interspecific interaction in community ecology. Compared with other regions in the world, Taiwan provides an excellent foundation for continued ecological investigations of *Ficus* species and their associated communities.

## Authors’ original submitted files for images

Below are the links to the authors’ original submitted files for images. Authors’ original file for figure 1
