# Supplementary figures and images for: Ficus (Moraceae) and fig wasps (Hymenoptera: Chalcidoidea) in Taiwan
Source: Bot Stud. 2015 May 16;56:11. doi: 10.1186/s40529-015-0090-x (PMC5432906; doi:10.1186/s40529-015-0090-x)

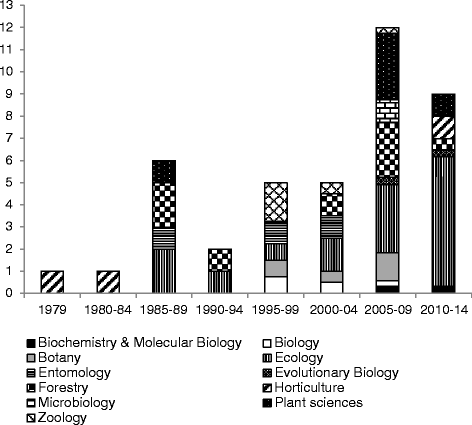

Supplement: Supplementary file 1 — Authors’ original file for figure 1 [file 40529_2015_90_MOESM1_ESM.gif]
